# Organophosphate or organochlorines or something else….?

**DOI:** 10.4103/0972-5229.53114

**Published:** 2009

**Authors:** Ritesh Aggarwal, Shekhar Diddee

**Affiliations:** **From:** Medical ICU, Aditya Birla Memorial Hospital, Chinchwad, Pune, India

**Keywords:** Deltamethrin, pyrethroids, status epilepticus

## Abstract

Deltamethrin belongs to the pyrethroids group of insecticides. Poisoning due to pyrethroids clinically resembles poisoning due to other common insecticides like organophosphates. This overlap of presentations can lead to misdiagnosis. We present here such a case of deltamethrin poisoning.

## Introduction

Deltamethrin poisoning is uncommon throughout the world. Insecticides containing pyrethroids are considered to have a relatively lower toxicity potential compared to other insecticides such as those containing organophosphates (OP). Interestingly there are several similarities (in clinical presentation) between pyrethroid poisoning, organophosphate and organochlorine poisoning. Physicians working in emergency departments or ICUs should be aware of this particular poisoning which can clinically mimic OP poisoning, because management of the two differs.

We present a case of acute deltamethrin poisoning, whose clinical presentation was more like organophosphate/organochlorine poisoning and quite unlike typical pyrethroid intoxication and she could have been easily misdiagnosed as OP poisoning.

## Case Report

A 16-year-old girl was admitted to the ICU with a history of consuming poison (20 ml of deltamethrin containing 12.5mg/ml, total dose = 250mg) following a domestic dispute. She presented with status epilepticus. As per the details gathered from the relatives, the patient was having continuous generalized tonic clonic seizures (GTCs) (status) since one hour prior to admission and was even convulsing during her transfer to the hospital. On arrival the patient was immediately intubated and ventilated and resuscitation started.

The convulsions were treated with injection Lorazepam, but there was no response. Further, doses of IV Midazolam/ Valproate/Phenobarbitone were given, but the convulsions continued. A bolus does of Thiopentone was then given, after which she stopped convulsing. She was started on a thiopentone infusion. Despite the thiopentone infusion, she had four episodes of GTCs in the next six hours, which were managed with further boluses of thiopentone (200mg) and increase in rate of the infusion. Inotropes were started and IV fluids were continued which were guided by CVP and arterial BP monitoring. Her rhythm on ECG was normal and lungs were clinically dry. CNS examination was not possible because she was sedated with thiopentone. Her pupils were dilated (6mm) and fixed.

An EEG showed 80% suppression of the brain. CT scan showed diffuse cerebral edema with effacement of sulci and chinking of lateral ventricles.

Lab reports showed normal S. cholinesterase levels (4340 IU/L), normal serum electrolytes and urine negative for myoglobin.

After 48 hours of being on thiopentone infusion (24 hours without convulsions), she was weaned off thiopentone infusion. The patient was triggering the ventilator well by the next day (day 4). Supports were hence taken off gradually. The patient's pupils became reactive by day 5.

In the following days, the patient was tracheostomized (in view of anticipated prolonged mechanical ventilation) and T-piece trials were initiated soon. By day 11, she was opening eyes to call and was moving all four limbs, but with reduced power. Tracheostomy was decannulated on day 14 [Table 1].

**Table 1:** Summary of events during the hospital stay

Days	Events
Day 1	On infusion thiopentone/Inotropes. Convulsions +
Day 3	Off thiopentone
Day 4	Triggering ventilator
Day 5	Off Inotropes
	Pupils reacting
	Responding to pain
Day 6- Day 8	Tracheostomy done
	T-piece trials given
Day 9	Extension to pain
Day 10	Flexion to pain
Day 11	Eye opening to call
	Moved all limbs to call
	Decreased (motor) power. Neck holding weak
Day 14	Tracheostomy decannulated. Neck holding good
Day 16	NCV studies (axonal degeneration)
Day 18	Shifted to ward
Day 21	Wheelchair ambulation
Day 26	Discharged

Nerve conduction studies were done in view of her poor muscle strength which showed “severe, generalized, predominantly motor axonal polyneuropathy”.

The patient was shifted to the ward after 18 days of ICU stay, on supportive care. Wheelchair ambulation was started on day 21 and the patient was discharged on day 26.

## Discussion

Synthetic pyrethroids are known to have a high insecticidal activity, low toxicity in mammals and no residue in biosphere.[1] Therefore, they have been used to control a wide variety of agricultural pests. At present, the most popularly used pyrethroids are deltamethrin, fenvalerate, and cypermethrin.

Acute human poisoning from exposure to these insecticides is rare; however, they can cause skin and upper airway irritation and hypersensitivity reactions. No clinical case of acute pyrethroid poisoning had ever been reported in literature until outbreaks of acute deltamethrin poisoning occurred in spraymen in China in 1982. After that, there have been reports of pyrethroid poisoning, but almost all of them are occupational poisoning.

Occupational poisoning occurs due to inappropriate handling (e.g., spraying with high concentration), long exposure duration, spraying against wind or lack of personal protection. The signs and symptoms occur due to exposure of skin, eyes, and upper airways to pyrethroids, and include burning, tingling sensation, numbness, parasthesias, lacrimation, photophobia, conjunctival congestion, and bronchospasm. Most of the symptoms/signs are not serious and resolve in 5-6 days.

Oral ingestion of pyrethroids leads to epigastric pain, nausea, vomiting, dizziness, headache, and fatigue. There are features of neuro-excitement like photophobia, parasthesias, twitching, and fasciculations.[2] Convulsions generally occur with consumption of doses above 500mg. The frequency of convulsions can be 10-30 times a day. Severe cases may be associated with pulmonary edema.

Management of pyrethroid poisoning involves supportive care and symptomatic management. Gastric lavage should be given. There is no specific antidote. Atropine should be given to decrease secretions in cases with salivation and pulmonary edema. Low doses of atropine (0.5-10mg) are generally sufficient in these cases.

Pyrethroid poisoning can be easily misdiagnosed as organophosphate or organochlorine poisoning. There are reported cases in literature where the patients were misdiagnosed as acute organophosphate poisoning.[3,4]

The similarities between the two include:

1. Smell of pyrethroids is somewhat similar to OP. (This is basically due to the common hydrocarbon solvents in which these agents are dispensed. These solvents impart the common properties of offensive smell, inflammability and airway irritation).
2. Muscle fasciculations and pulmonary edema can occur in both.
3. Convulsions can occur in both.

To differentiate between these two kinds of pesticide poisonings, exposure history is most important. Moreover, there is no inhibition of plasma cholinesterase in pyrethroid poisoning and requirement of atropine is usually less than 10mg [Table 2]. Pyrethroid poisoning is associated with better prognosis even in seriously affected patients.

**Table 2:** Comparison between organophosphates, organochlorines, and pyrethroids

	Organophosphates	Organochlorines	Pyrethroids
Examples	Malathion/Parathion	DDT/BHC	Deltamethrin
Mechanism of action	Anti-cholinesterase	Hyper excitability of nervous system	Neuro-excitation by blocking Na^+^ channels
Clinical features	√ Excessive salivation	√ Neurological signs and symptoms	√ Neurological signs and symptoms
	√ Sweating	√ Giddiness/ parasthesias	
	√ Pin point pupil Fatigue/giddiness	√ Twitching/Tremors	√ Tingling/numbness
	√ Muscle twitching/ N-M weakness	√ Hallucinations	√ Fasciculations
Convulsions	Rare	+++	+
Pulm Edema	Common	Rare	Rare
Antidote	PAM[5]	None	None
Atropine requirements	High	Moderate	Low
S. Cholinesterase	Low	Normal	Normal
Prognosis	Bad	Better	Good
